# Integrating Experimental and Computational Approaches to Cardioprotection: Vascular Reactivity, Molecular Docking, and ADMET Modeling of *Melicoccus bijugatus* (Guinep)

**DOI:** 10.3390/ijms262010228

**Published:** 2025-10-21

**Authors:** Keaton Logan, Javier Palacios, Sussan Lopez, Wesley Gray, Chukwuemeka R. Nwokocha

**Affiliations:** 1 Department of Applied Sciences, Faculty of Engineering and Applied Technology, Caribbean Maritime University, Main Campus, Palisadoes Park, Norman Manley Highway, Kingston P.O. Box 8081, Jamaica; klogan@faculty.cmu.edu.jm; 2 Carrera de Química y Farmacia, Facultad de Ciencias de la Salud, Universidad Arturo Prat, Iquique 1110939, Chile; sussan.j.lopez.m@gmail.com; 3 Department of Environmental Toxicology, Southern University and A&M College, Baton Rouge, LA 70807, USA; wesley_gray@subr.edu; 4 Department of Basic Medical Sciences Physiology Section, Faculty of Medical Sciences, The University of the West Indies, Mona, Kingston 7, Jamaica

**Keywords:** *Melicoccus bujugatus*, vasodilation, ADMET, molecular docking, cardio protection

## Abstract

*Melicoccus bijugatus* (Guinep) is traditionally consumed in the Caribbean and Latin America for its health benefits, yet its cardiovascular effects remain underexplored. This study investigated the therapeutic potential of Guinep by combining experimental and computational approaches. The biological evaluation of the Guinep extract was conducted by assessing the effects of modulating Angiotensin-Converting Enzyme (ACE), Angiotensin II Type 1 Receptor (AT1R), and Voltage-Gated Calcium Channels (VGCC) on vascular reactivity. Metabolites previously identified by high-resolution UHPLC-Q-Orbitrap mass spectrometry were further examined using in silico tools, including ADMET (Absorption, Distribution, Metabolism, Excretion, and Toxicity) prediction (pkCSM), biological activity prediction (PASS server), and molecular docking (AutoDock Vina) against cardiovascular targets (ACE: PDB 1O86, AT1R: PDB 4ZUD, VGCC: PDB 8WE8). Docking results revealed that phytochemicals such as isorhamnetin-3-O-glucoside and 3-O-caffeoylquinic acid displayed strong binding affinities with ACE (−9.3 and −8.5 kcal/mol), AT1R (−8.2 and −7.6 kcal/mol), and VGCC (−8.6 and −7.6 kcal/mol), in several cases matching or surpassing standard antihypertensive drugs. Key hydrogen bond interactions closely resembled those of reference ligands, suggesting pharmacophoric similarity. ADMET modeling confirmed favorable pharmacokinetic profiles and low predicted toxicity, supporting their drug-like potential. These findings are consistent with in vivo evidence of Guinep’s hypotensive, antioxidant, and vasodilatory properties. Vascular relaxation of Guinep extract was predominantly mediated by blockade of VGCC (53%) and AT1R (48%), while ACE inhibition accounted for 24%. Collectively, the results demonstrate that Guinep contains bioactive phytochemicals with multitarget cardiovascular activity, particularly as ACE, AT1R, and VGCC modulators. This study validates the traditional use of Guinep.

## 1. Introduction

Guinep (*Melicoccus bijugatus* Jacq.), also known as Spanish lime or mamoncillo, is a tropical fruit traditionally used in folk medicine. It is used in the management of hypertension, asthma, diarrhea and constipation. Emerging evidence suggests that it may offer cardiovascular benefits, primarily through its antioxidant and anti-inflammatory activities [1]. Guinep fruit pulp extract protected rat hearts from isoproterenol-induced damage, primarily through its antioxidant and anti-inflammatory activities, with high-resolution mass spectrometry identifying key phenolic metabolites such as cirsimaritin and quercetin derivatives. In a separate study, the aqueous extract of Guinep demonstrated both hypotensive and antihypertensive effects in rats, acting mainly via the blockade of vascular calcium channels. The identified bioactive metabolites are therefore implicated in both the observed cardioprotective and blood pressure-lowering mechanisms of action [2,3]. Caffeic acid, a constituent of Guinep is reported to Inhibits vascular smooth muscle cell proliferation induced by angiotensin II in stroke-prone hypertensive rats [4], it is also a selective inhibitor for the biosynthesis of leukotrienes, or compounds that sustain inflammatory reactions [5]. The fruit also contains Resveratrol, which is a broad-spectrum nonspecific inhibitor of the activation of NFκB in several cell lines [6], while coumaric acid, a common dietary phenol, inhibits platelet activity in vitro and in vivo [7].

The identified flavonoids present in Guinep include hydroxycinnamic acid, sinapic acid epicatechin, catechin, epigallocatechin, B-type procyanidins (dimers), naringenin, naringenin derivatives, phloretin, phloridzin, quercetin, myricetin and resveratrol [7]. Related fruits with high flavonoid content have been shown to enhance nitric oxide (NO) bioavailability, improving vascular relaxation and lowering blood pressure [2,3,8]. Guinep is useful in the treatment of gastrointestinal problems due to constituents like epicatechin catechin and procyanidin B2, which inhibit chloride transport by the cystic fibrosis transmembrane conductance regulator (CFTR) in human colon epithelial cell [6,9,10].

Vascular reactivity experiments were conducted to evaluate the biological effects of the extract, focusing on its modulation of Angiotensin-Converting Enzyme (ACE), Angiotensin II Type 1 Receptor (AT1R), and Voltage-Gated Calcium Channels (VGCC). The integration of UHPLC-Q-Orbitrap mass spectrometry for compound identification, spectral activity prediction using the PASS server, ADMET modeling, and molecular docking provides a comprehensive strategy to investigate and validate the cardiovascular potential of *M. bijugatus*. This combined experimental and computational approach highlights its promise in natural product–based drug discovery and the development of novel antihypertensive agents [11,12]. UHPLC–MS data from our previous study [3] helped identify several bioactive constituents in *M. bijugatus* (Guinep). Among these, Isorhamnetin-3-O-glucoside and 3-O-caffeoylquinic acid were selected because they belong to flavonoid and phenolic acid classes, which are widely recognized for their antihypertensive [13,14] and ACE-inhibitory properties [15]. Docking allowed virtual screening of Guinep compounds against cardiovascular target proteins like Angiotensin-converting enzyme (ACE) which regulates blood pressure, to predict binding affinity (kcal/mol), Hydrogen bonds and interaction and possible mechanisms of action [16,17]. This integrative method will help to further validate traditional uses of Guinep with scientific evidence, prioritize lead compounds for further in vitro or in vivo testing and guide formulation of functional foods or nutraceuticals.

## 2. Results

### 2.1. Effect of M. bijugatus on Contractile Response

Angiotensin-converting enzyme (ACE), angiotensin II type 1 receptor (AT1R), and voltage-gated calcium channels may be involved in the vascular response. We chose vascular response experiments because they offer a simple approach to comparing the function of the renin–angiotensin–aldosterone system (RAAS), the involvement of ACE, and ATR1. They could even allow for comparisons of the function of voltage-gated calcium channels, all of which are key to underpinning blood pressure regulation.

**Figure 1:**
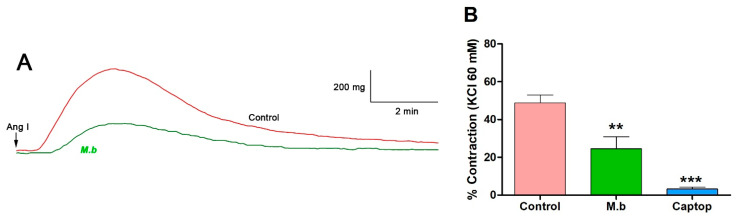
Original trace showing effect of *M. bijugatus* (M.b.) on contractile response to Ang I (**A**). The responses are expressed as % of maximum KCl-induced contraction in intact rat aorta. The tissues were pre-incubated with *M. bijugatus* (400 μg/mL) for 20 min before adding Ang I (10^–7^ M). Captopril (Captop; 10^–5^ M) was used as an inhibitor of ACE (**B**). Each data point represents the mean ± SEM. ** *p* < 0.01, *** *p* < 0.001 vs. Control; *n* = 5–7.

To evaluate the activity of the ACE on the vascular response in the presence of *M. bijugatus* (M.b.), contraction was induced with angiotensin I (Ang I). The results indicated that preincubation of intact rat aorta (with vascular endothelium), the extract reduced contraction to Ang I: 49% ± 4 control versus 25% ± 6 M.b. (*p* < 0.01). While captopril was significantly (*p* < 0.001) lower, 3% ± 1 (Figure 1).

**Figure 2:**
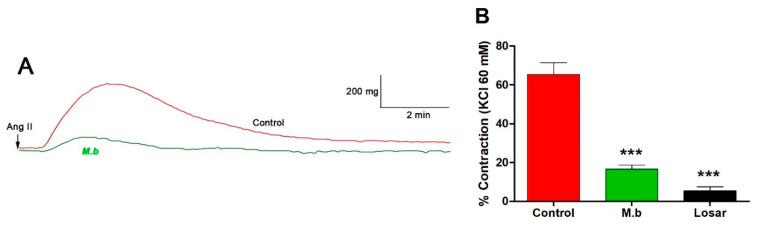
Original trace showing effect of *M. bijugatus* (M.b.) on contractile response to Ang II (**A**). The responses are expressed as % of maximum KCl-induced contraction in intact rat aorta. The tissues were pre-incubated with *M. bijugatus* (400 μg/mL) for 20 min before adding Ang II (10^–7^ M). Losartan (Losar; 10^–5^ M) was used as a blocker of ATR1 (**B**). Each data point represents the mean ± SEM. *** *p* < 0.001 vs. Control; *n* = 5–7.

The role of the angiotensin II receptor (AT1R) in vascular response was assessed by inducing contraction with angiotensin II (Ang II) in the presence of *M. bijugatus* extract. Preincubation of intact rat aorta with the extract significantly reduced Ang II-induced contraction compared to control: 65% ± 6 control versus 17% ± 2 M.b. (*p* < 0.01). While captopril was significantly (*p* < 0.001) lower, 5% ± 2 (Figure 2).

**Figure 3:**
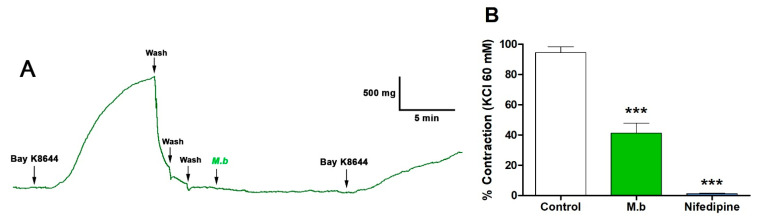
Original trace showing effect of *M. bijugatus* (Mb) on contractile response to Bay K8644 (**A**). The responses are expressed as % of maximum KCl-induced contraction in intact rat aorta. The tissues were pre-incubated with *M. bijugatus* (400 μg/mL) for 20 min before adding Bay K8644 (10^–8^ M). Nifedipine (10^–6^ M) was used as a calcium channel blocker (**B**). Each data point represents the mean ± SEM. *** *p* < 0.001 vs. Control; *n* = 5–7.

The involvement of voltage-gated calcium channels in vascular response was evaluated by inducing contraction using the voltage-gated calcium channel agonist Bay K8644 in the presence of *M. bijugatus* extract. Preincubation of intact rat aorta with the extract resulted in a significant reduction in BayK8644-induced contraction compared to the control group: 94% ± 4 control versus 41% ± 7 M.b. (*p* < 0.01). While nifedipine was significantly (*p* < 0.001) lower, 1% ± 0.3 (Figure 3).

### 2.2. Redocking Validation

**Figure 4:**
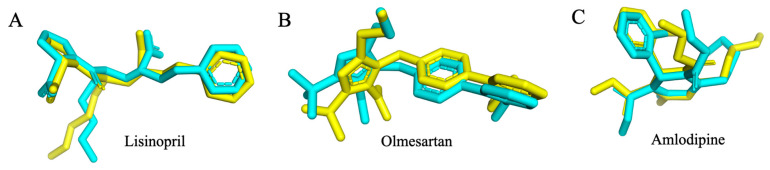
Validation of docking protocol showing RMSD: 1.201 Å for ACE–lisinopril (**A**), 0.617 Å for AT1R–Olmesartan (**B**) and 1.294 Å for VGCC–amlodipine (**C**). (Yellow is the co-crystallized pose, and blue is the re-docked pose).

The docking protocol accurately reproduced the crystallographic poses of the reference inhibitors in all targets, with RMSD values of 1.201 Å for ACE–lisinopril, 0.617 Å for AT1R–Olmesartan, and 1.294 Å for VGCC–amlodipine. These results, all well within the accepted cutoff of <2.0 Å, confirm the robustness of the docking setup applied to the phytochemical dataset Figure 4.

### 2.3. Binding Affinity and Docking Pose Analysis

**Figure 5:**
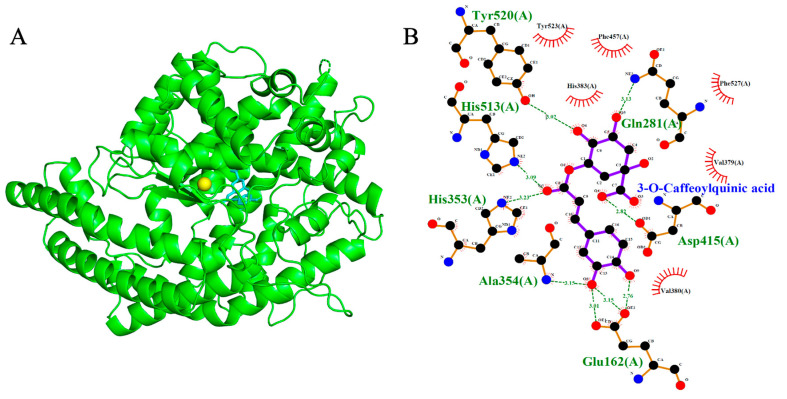
Models showing the potential pose of 3-O-Caffeoylquinic acid in the active site of ACE. (**A**) The green molecule is representative of the enzyme ACE, while the blue molecules represent the ligand and the yellow sphere represents a Zn atom. (**B**) The binding mode of ACE residues with 3-O-Caffeoylquinic acid (black dots with purple connecting lines) after docking at the ACE active site. Green dotted lines signify the formation of molecular forces (strong hydrogen bonds).

**Figure 6:**
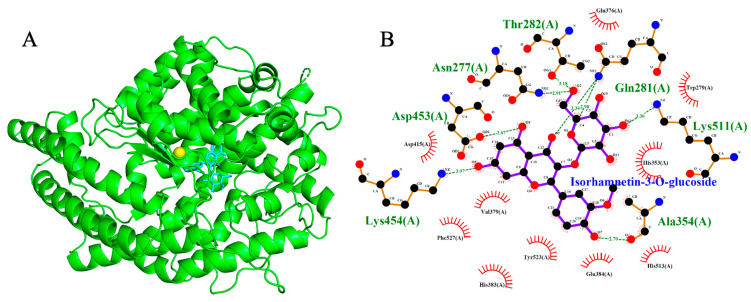
Models showing the potential pose of Isorhamnetin-3-O-glucoside in the active site of ACE. (**A**) The green molecule is representative of the enzyme ACE, while the blue molecules represent the ligand and the yellow sphere represents a Zn atom. (**B**) The binding mode of ACE residues with Isorhamnetin-3-O-glucoside (black dots with purple connecting lines) after docking at the ACE active site. Green dotted lines signify the formation of molecular forces (strong hydrogen bonds).

**Figure 7:**
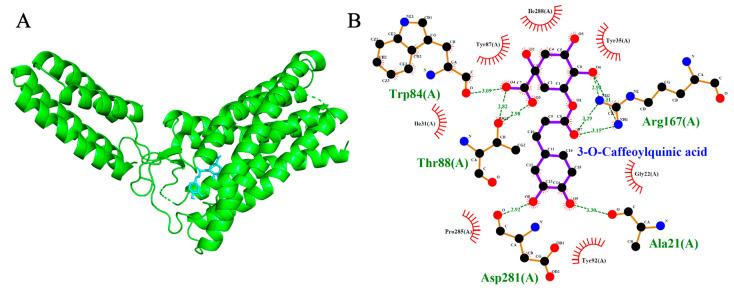
Models showing the potential pose of 3-O-Caffeoylquinic acid in the active site of AT1R. (**A**) The green molecule is representative of the receptor AT1R, while the blue molecules represent the ligand. (**B**) The binding mode of AT1R residues with 3-O-Caffeoylquinic acid (black dots with purple connecting lines) after docking at the AT1R active site. Green dotted lines signify the formation of molecular forces (strong hydrogen bonds).

**Figure 8:**
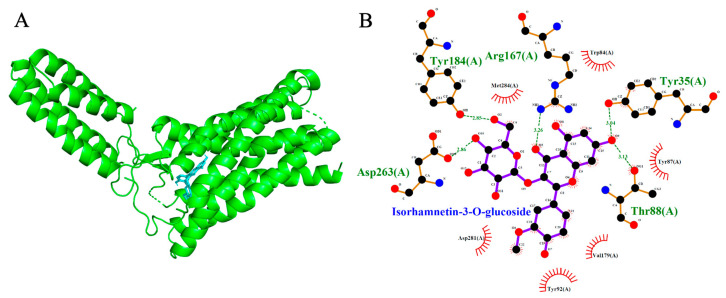
Models showing the potential pose of Isorhamnetin-3-O-glucoside in the active site of AT1R. (**A**) The green molecule is representative of the receptor AT1R, while the blue molecules represent the ligand. (**B**) The binding mode of AT1R residues with Isorhamnetin-3-O-glucoside (black dots with purple connecting lines) after docking at the AT1R active site. Green dotted lines signify the formation of molecular forces (strong hydrogen bonds).

**Figure 9:**
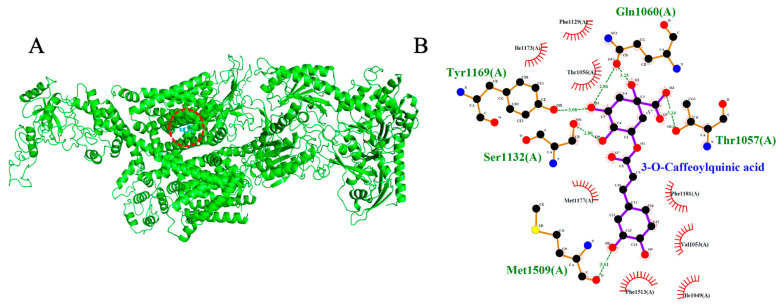
Models showing the potential pose of 3-O-Caffeoylquinic acid in the active site of VGCC. (**A**) The green molecule is representative of the receptor VGCC, while the blue molecules represent the ligand (highlighted by a red dotted circle). (**B**) The binding mode of VGCC residues with 3-O-Caffeoylquinic acid (black dots with purple connecting lines) after docking at the VGCC active site. Green dotted lines signify the formation of molecular forces (strong hydrogen bonds).

**Figure 10:**
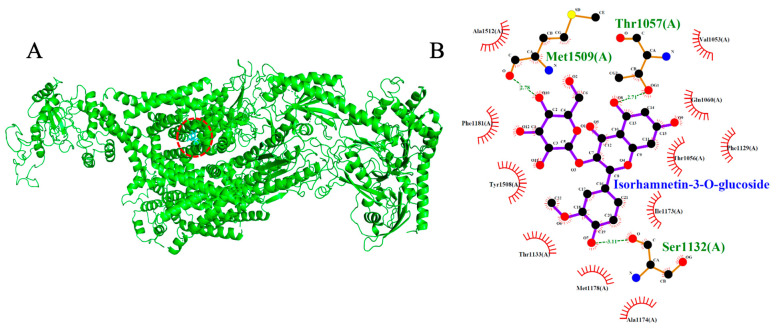
Models showing the potential pose of Isorhamnetin-3-O-glucoside in the active site of VGCC. (**A**) The green molecule is representative of the receptor VGCC, while the blue molecules represent the ligand (highlighted by a red dotted circle). (**B**) The binding mode of VGCC residues with Isorhamnetin-3-O-glucoside (black dots with purple connecting lines) after docking at the VGCC active site. Green dotted lines signify the formation of molecular forces (strong hydrogen bonds). Strongs hydrogen bonds were observed between the molecule and the Calcium ion (Ca^2+^) (pink molecule) at the active site of VGCC.

Molecular docking simulations were performed to evaluate the binding interactions of Isorhamnetin-3-O-glucoside and 3-O-Caffeoylquinic acid with three cardiovascular-related targets: ACE (1O86), AT1R (4ZUD), and VGCC (8WE8). Reference compounds (Lisinopril, Captopril, Losartan, Amlodipine, Nifedipine, Verapamil, and Diltiazem) were included to benchmark the binding energies and interaction profiles of the test ligands. Docking scores are summarized in Table 1, Table 2 and Table 3, with 2D and 3D interaction diagrams presented in Figure 5, Figure 6, Figure 7, Figure 8, Figure 9 and Figure 10.

### 2.4. Angiotensin-Converting Enzyme (ACE, PDB: 1O86)

Isorhamnetin-3-O-glucoside and 3-O-Caffeoylquinic acid demonstrated strong binding affinities toward ACE, with docking scores of −9.2 kcal/mol and −8.4 kcal/mol, respectively- exceeding those of the standard inhibitors Lisinopril (−8.2 kcal/mol) and Captopril (−5.6 kcal/mol). As expected, Lisinopril and Captopril adopted canonical binding poses near the Zn^2+^ ion in the ACE active site. Captopril formed three hydrogen bonds with Gln281, Lys511 and Tyr520, while Lisinopril formed four hydrogen bonds with Tyr523, Ala354, Glu384 and His513. The test ligands also docked within the Zn^2+^-coordinated binding region, albeit with distinct orientations compared to the reference compounds. Isorhamnetin-3-O-glucoside formed seven hydrogen bonds with Asp453, Lys454, Ala354, Gln281, Lys511, Asn277 and Thr282. Similarly, 3-O-Caffeoylquinic acid engaged in seven hydrogen bonds with Asp415, Tyr520, His513, His353, Ala354, Glu162 and Gln281, indicating strong anchoring within the catalytic pocket. Notably, both phytochemicals shared key binding residues with the standard inhibitors, suggesting structural mimicry and potential pharmacophoric relevance. Isorhamnetin-3-O-glucoside overlapped with Lisinopril at Ala354; also, with Captopril at Gln281 and Lys511 reflecting a highly similar interaction profile. Meanwhile, 3-O-Caffeoylquinic acid shared the Gln281 and Tyr520 binding site with Captopril; also, with lisinopril at His513 and Ala354. These overlaps reinforce the possibility that the phytochemicals could inhibit ACE via mechanisms analogous to established inhibitors, highlighting their potential therapeutic relevance.

### 2.5. Angiotensin II Type 1 Receptor (AT1R, PDB: 4ZUD)

Docking simulations revealed that both phytochemicals exhibited binding affinities to AT1R that were comparable to the reference antagonist Losartan. Isorhamnetin-3-O-glucoside and 3-O-Caffeoylquinic acid recorded binding energies of −8.2 kcal/mol and −7.5 kcal/mol, respectively, while Olmesartan and Losartan showed a slightly higher affinities at −8.8 kcal/mol. Docking of Olmesartan and Losartan reproduced their native binding poses, forming one hydrogen bond with Thr88 and three hydrogen bonds with Cys180, Tyr87 and Thr88, respectively. Isorhamnetin-3-O-glucoside established five hydrogen bonds with Tyr184, Asp263, Thr88, Tyr35 and Arg 167, reflecting a stable interaction profile. Notably, the shared interaction with Thr88 between Olmesartan, Losartan and Isorhamnetin-3-O-glucoside suggests a potential structural mimicry at a key anchoring site within the binding pocket. The molecule 3-O-Caffeoylquinic acid formed five hydrogen bonds with Trp84, Tyr88, Asp281, Ala21 and Arg167, also sharing binding interactions with Olmesartan and Losartan at Thr88. While occupying a distinct spatial orientation, it nonetheless targeted critical residues within the AT1R active site. The recurring involvement of these amino acids in interactions with the ligands highlights their significance as a conserved and essential binding residue for ligand recognition and stabilization.

### 2.6. Voltage-Gated Calcium Channel (VGCC, PDB: 8WE8)

Both test ligands exhibited binding affinities for VGCC that were comparable to those of established calcium channel blockers. Isorhamnetin-3-O-glucoside and 3-O-caffeoylquinic acid showed docking scores of −8.7 kcal/mol and −7.7 kcal/mol, respectively, which were similar to the reference drugs amlodipine (−6.4 kcal/mol), nifedipine (−6.9 kcal/mol), verapamil (−7.5 kcal/mol), and diltiazem (−8.1 kcal/mol). Among the reference compounds, amlodipine, nifedipine and diltiazem engaged primarily in hydrophobic interactions without forming hydrogen bonds. In contrast verapamil formed two hydrogen bonds, interacting with Thr1056 and Ser1132. Likewise, isorhamnetin-3-O-glucoside formed a network of three hydrogen bonds with Thr1057, Ser1132, and Met1509, indicative of a stable and well-anchored pose. 3-O-caffeoylquinic acid established five hydrogen bonds with Tyr1169, Ser1132, Met1509, Thr1057, and Gln1060, suggesting a deep and stable insertion into the transmembrane cavity. Notably, the shared interaction at Ser1132 between the test ligands and reference blocker verapamil imply potential mimicry of native ligand-binding behavior, reinforcing their possible role as calcium channel modulators.

### 2.7. ADMET Pharmacokinetic Properties

During drug discovery and development processes, both pharmacokinetic properties and toxicity play a critical role. These parameters not only determine the efficacy and safety of compounds, but are also crucial in assessing their environmental impact and human health. Several software programs used for drug discovery initially focus on identifying promising compounds that bind to the target protein of interest, as is the case with molecular docking. However, although the potency of a drug candidate compound is a key factor in the initial phase, in later stages, pharmacokinetic properties and toxicity determine whether this compound has efficacy and a safe therapeutic profile. In this study, the ADMET parameters (absorption, distribution, metabolism, excretion and toxicity) of the identified compounds of the Guinep fruit pulp extract and ACE inhibitor drugs were analyzed using the online tool pkCSM, which is based on graphical modeling that represents the different atoms and covalent bonds of a structure, along with their physicochemical properties, where structural patterns could be sought based on this data [18]. The six compounds proved ideal for conducting a study aimed at predicting pharmacokinetic properties, since in the preliminary molecular docking study, this group of molecules identified in guinep pulp [3] showed a lower score and, therefore, a higher affinity with the therapeutic target ACE, ATR1, and VGCC. The results are presented in Table 4.

### 2.8. Biological Activity Based on Spectra

Computer-aided biological activity prediction is one of the most efficient and cost-effective methods for screening a series of ligands, allowing the most promising lead compounds to be identified [19]. The metabolites present in Guinep extract underwent comparative analysis and efficient interpretation of the extensive biomedical and clinical information available on drugs already studied and registered in the PASSonline, https://way2drug.com/PassOnline/ (accessed on 23 July 2025). Table 5 shows the results obtained for the compounds identified in the Guinep extract, using amlodipine, nifedipine, captopril, diltiazem, losartan, and verapamil as reference drugs.

## 3. Discussion

This study integrated biological evaluation of the *M. bijigatus* extract with previously reported UHPLC–MS data [3], ADMET, biological activity based on spectra and Docking Insights. The vascular reactivity assay was employed to evaluate the contribution of Angiotensin-Converting Enzyme (ACE), Angiotensin II Type 1 Receptor (AT1R), and Voltage-Gated Calcium Channels (VGCC) to the vasorelaxant effects of *M. bijugatus* extract. The findings revealed that vascular relaxation (calculated as the difference between contractile response to Ang I in absence or presence of *M. bijugatus*) was mediated predominantly through AT1R blockade (48%), while ACE inhibition accounted for approximately 24% of the observed effect. In a previous study, we further demonstrated that *M. bijugatus* extract significantly reduced blood pressure in hypertensive models, including DOCA-salt and L-NAME–induced hypertensive rats, supporting its antihypertensive potential [2]. Since both hypertensive models involve dysregulation of the Renin–Angiotensin–Aldosterone System (RAAS) [20,21], the inhibitory effect of the extract on ACE and its blocking action on AT1R could account for the observed reduction in blood pressure. Furthermore, the inhibition of VGCC by the extract in vascular tissue is consistent with its vasorelaxant effects observed in intact rat aorta, which are endothelium-dependent and mediated via nitric oxide [2]. In the present study, the contribution of VGCC blockade to vascular relaxation was comparable to that of AT1R inhibition, accounting for approximately 53% of the overall effect.

UHPLC–MS analysis from our previous study [3] identified two lead compounds: Isorhamnetin-3-O-glucoside and 3-O-caffeoylquinic acid which possess established hypotensive effects while ADMET confirmed Favorable GI absorption (>30%) and low toxicity and Low BBB penetration minimizes CNS side effects. We evaluated the molecular interactions of two naturally occurring phytochemicals, Isorhamnetin-3-O-glucoside and 3-O-Caffeoylquinic acid, with three key cardiovascular targets: angiotensin-converting enzyme (ACE1), angiotensin II type 1 receptor (AT1R), and voltage-gated calcium channel (VGCC). This was done to assess their potential as multi-target agents in antihypertensive therapy. The molecular docking simulations revealed that both phytochemicals showed significant binding affinity to all three receptors, often matching or surpassing those of established pharmaceutical inhibitors. For ACE, Isorhamnetin-3-O-glucoside and 3-O-caffeoylquinic acid exhibited binding energies of −9.2 kcal/mol and −8.4 kcal/mol, respectively, compared to −8.2 kcal/mol for Lisinopril and −5.6 kcal/mol for Captopril. The stronger binding affinities of both compounds relative to the reference inhibitors suggest a high potential for ACE inhibition, consistent with their predicted hydrogen-bonding and hydrophobic interactions within the active site. These findings highlight the ability of naturally derived compounds to interact favorably with the catalytic pocket of ACE, comparable to clinically used inhibitors. Similarly, for VGCC, Isorhamnetin-3-O-glucoside and 3-O-Caffeoylquinic acid demonstrated binding energies of −8.7 kcal/mol and −7.7 kcal/mol respectively, which were comparable and, in some cases, outperforming known VGCC antagonist: Amlodipine (−6.4 kcal/mol), Nifedipine (−6.9 kcal/mol), Verapamil (−7.5 kcal/mol), and Diltiazem (−8.1 kcal/mol). In the case of AT1R, Isorhamnetin-3-O-glucoside and 3-O-Caffeoylquinic acid recorded binding energies of −8.2 kcal/mol and −7.5 kcal/mol, respectively, closely approximating that of Olmesartan (−8.8 kcal/mol) and Losartan (−8.8 kcal/mol), a widely recognized AT1R antagonist. This level of interaction suggests competitive binding potential that may have therapeutic implications, as echoed in earlier reports identifying natural polyphenols as viable AT1R modulators [19,20,21].

Beyond their binding strength, the analysis revealed several conserved residues between the phytochemicals and standard inhibitors, indicating structural and functional mimicry.

In the ACE binding pocket, Isorhamnetin-3-O-glucoside formed hydrogen bonds with Gln281, Ala354 and Lys511, residues also frequently targeted by the well-known inhibitors Lisinopril and Captopril. Conversely, 3-O-Caffeoylquinic acid engaged Gln281, His513, Ala354 and Tyr520, mimicking key anchor residues involved in Lisinopril and Captopril binding. These interactions are supported by docking studies demonstrating the importance of these residues in ACE inhibition, particularly by phenolic or flavonoid-based natural compounds. For instance, Zheng et al. (2019) noted similar amino acid binding points like Gln281, His353, Asp415 and His513 (residues in S2 binding pocket of ACE) which serve as common interaction points for polyphenol-derived inhibitors in ACE, thereby validating the potential mimicry observed in this study [22]. At the AT1R site, Isorhamnetin-3-O-glucoside formed hydrogen bonds with Thr88, which was also involved in Olmesartan and Losartan binding. This overlap with Thr88 suggests a pharmacophoric similarity. 3-O-Caffeoylquinic acid, while adopting a distinct orientation, targeted multiple critical residues within the active site highlighting a strong binding profile that aligns with key anchoring residues of AT1R inhibitors. Of note, an overlap was recorded with Thr88 residue as also observed for Olmesartan and Losartan. Key binding interactions between Trp84, Tyr35 and Arg167 and our ligands were also recorded (Table 2). These findings are consistent with a recent docking and molecular dynamics study by Mahmoud et al. (2024), which emphasized similar amino acids as central hydrogen bonding residues for natural flavonoid- and polyphenol-based AT1R inhibitors, reinforcing their functional role in antagonist binding [23]. In VGCC docking, Isorhamnetin-3-O-glucoside and 3-O-Caffeoylquinic acid formed three and five strong hydrogen bonds, respectively, including a shared interaction at Ser1132 between the reference blocker (verapamil) suggesting the potential to mimic native calcium antagonists. These findings align with molecular docking models that describe the critical importance of polar and acidic residues for stable VGCC inhibition [24].

The in silico findings are strongly supported by prior in vitro and in vivo evidence of cardiovascular activity. Flavonoid glycosides such as Isorhamnetin-3-O-glucoside are known to hydrolyze to their aglycone forms (Isorhamnetin) before absorption, a process observed for other glycoside derivatives [25]. Isorhamnetin, the biologically comparable aglycone of Isorhamnetin-3-O-glucoside has demonstrated direct inhibition of VGCC and reduction in intracellular Ca^2+^ levels in rabbit and rat vascular smooth muscle cells [26]. In isolated thoracic aorta preparations, it induced dose-dependent vasodilation and hypotension, attributed to both calcium antagonism and potential nitric oxide (NO)-mediated mechanisms [27]. Additional studies have confirmed Isorhamnetin’s antihypertensive effect through endothelium-dependent pathways and endothelial nitric oxide synthase (eNOS) activation [28]. Similarly, 3-O-Caffeoylquinic acid, a major chlorogenic acid isomer, has been widely studied for its ACE inhibitory activity, blood pressure-lowering effects, and vascular protection. In spontaneously hypertensive rats, it significantly attenuated systolic pressure and improved endothelial function via NO bioavailability [29]. Recent work by Nguyen et al. (2024) also confirmed its hypotensive activity, reinforcing its pharmacological alignment with the docking results observed [29]. Collectively, these findings suggest that Isorhamnetin-3-O-glucoside and 3-O-Caffeoylquinic acid have the potential to function as multi-target cardiovascular modulators, simultaneously engaging ACE, AT1R, and VGCC. Such poly-pharmacological activity could be advantageous in the treatment of multifactorial conditions like hypertension and heart failure, where targeting multiple pathways offers greater efficacy and lower resistance development. Additionally, their structural similarity to reference drugs, evidenced by shared binding residues and pose overlap, supports their candidacy for lead optimization in future drug development pipelines. Our multi-target mechanism studies using molecular docking thus showed a shared binding residues (Gln281) with reference drugs for ACE inhibition, 3 H-bonds by Isorhamnetin-3-O-glucoside vs. 0–1 in standard antagonists for VGCC blockade and a polypharmacological potential through a simultaneous ACE/AT1R/VGCC modulation, which can address hypertension synergistically.

The ADMET analysis revealed that the 6 compounds studied have good cellular penetrability with molecular weights ranging from 300.26 to 478.41 g/mol, since values below 500 g/mol are usually within the optimal range for absorption [18]. However, all compounds identified in the extract had Caco-2 permeability values below 0.52, including lisinopril, the reference drug. Nevertheless, aflavarin (0.44) was the only compound that showed a greater possibility of having a good apparent permeability, as did the drug captopril (1.16) [30], but with intestinal absorption ranging from 29.19% to 92.22%, suggesting that they may be slowly absorbed in the small intestine, which could be attributed to intestinal/microbial deglycosylation that releases the more permeable aglycone and thus reduces the intestinal permeability of the remaining structure of the glycoside metabolites. However, it should be noted that aflavarin has excellent gastrointestinal absorption, even superior to that of the drugs captopril and Lisinopril (Table 4). In terms of distribution, all compounds have a steady-state volume of distribution (VDss) < −0.15, which means that they may have low tissue distribution and therefore may be limited to remaining in plasma. Regarding the ability to cross the blood–brain barrier (BBB), the compounds have logBB values < 1.3, which indicates that they are unlikely to penetrate the blood–brain barrier. In addition, the CNS permeability values obtained for these compounds are below −2 [18], which supports the previous result. Regarding metabolism, it is observed that *Salicylic acid glucoside* and *Coumaric acid glucoside* do not inhibit the CYP3A4 isoform, compared to the other compounds, which indicates an acceptable hepatic biotransformation. In terms of excretion, the compounds aflavarin and Isorhamnetin-3-O-glucoside showed greater renal clearance, since they are within the acceptable total clearance range of 0.5–1.5, indicating a more efficient elimination compared to the compounds of the Guinep pulp extract. In contrast, the other compounds have lower values, which suggests a possible difficulty in their elimination, which could prolong their half-life and potentially increase their risk of accumulation in the body, including the ACE inhibitors analyzed. In terms of toxicity, the values of acute oral toxicity in rats (LD_50_) ranged from 1.46 to 2.45 mol/kg. According to OECD test methods, compounds with an LD_50_ greater than 2 mol/kg [18] are considered to have low chronic toxicity. Although not all compounds meet this threshold, data suggest that their toxicity is relatively low, in particular the compound Salicylic acid glucoside and aflavarin. The values of chronic oral toxicity in rats (LOEL) are between 1.80 and 3.67, they are values >1 [18], therefore they would represent a low chronic toxicity. Therefore, these results indicate that the compounds identified in the pulp extract of Melicoccus bujugatus have an acceptable pharmacokinetic profile, with low to moderate gastrointestinal absorption, acceptable metabolism, moderate elimination, although some compounds may require evaluation of their potential for accumulation, and, finally, low toxicity.

The prediction of the biological activity of the metabolites identified in the Guinep pulp extract showed that the compounds isorhamnetin-3-O-glucoside, 3-O-caffeoylquinic acid, salicylic acid glucoside, coumaric acid glucoside, feruloyl glucoside, and aflavarin could have cardioprotective activity, with isorhamnetin-3-O-glucoside (0.988) and salicylic acid glucoside (0.698) standing out as having a high probability of cardioprotective activity. Similarly, these compounds have vasoprotective activity with a high probability, with the exception of aflavarin, which is related to a study on the pharmacological effects of isorhamnetin as a prophylactic for cardiovascular diseases [31]. Likewise, a study showed that salicylic acid contributes to a cardioprotective effect at the level of cardiac mitochondrial function [32]. Regarding the coronary vasodilator effect, it was observed that all compounds had this activity, with the metabolite isorhamnetin-3-O-glucoside (0.666) and feruloyl glucoside (0.661) standing out. At the same time, all identified metabolites of Guinep extract also have a peripheral vasodilatory effect, with the exception of aflavarin, highlighting the compounds feruloyl glucoside (0.724) and coumaric acid glucoside (0.696), both of which are more likely to have pharmacological action as peripheral vasodilators. This could be related to a study that showed that ferulic acid improved acetylcholine-induced endothelium-dependent vasodilation in spontaneously hypertensive rats [33]. In addition, in one study, p-coumaric acid showed a moderate endothelium-dependent vasodilator effect [34]. With regard to the calcium-regulating effect, it was demonstrated that all Guinep metabolites are likely to act at this level, with 3-O-caffeoylquinic acid (0.58) and coumaric acid glycoside (0.405) standing out as having a higher probability of activity than the other metabolites. However, no metabolite demonstrated calcium channel blocking activity, which is inconsistent with the molecular docking results for VGCC, where affinities superior to dihydropyridines and other calcium channel blockers were identified (Table 3). Therefore, we recognize that PASS may reflect the limitations of the chemical space trained by PASS for this class; thus, we prioritize functional evidence (“Bay K8644” in the aorta) and DHP cavity-focused docking. In future work, we will incorporate “molecular dynamics (MD) + MM/GBSA” to strengthen the inference in VGCC.

Guinep compounds outperform or match FDA-approved drugs in binding affinity while maintaining safety (low hepatotoxicity, LD_50_ > 1.46 mol/kg), and further validates our earlier In vitro/vivo studies [2] on the efficacy and use of *M. Bujugatus* for cardiovascular management because of its Competitive binding to ACE, AT1R, and VGCC (showing strong target engagement), and Optimal ADMET profiles for lead development, in these way, validating Guinep’s traditional use and supports its potential in nutraceuticals [2] or antihypertensive adjuvants [2].

## 4. Materials and Methods

### 4.1. Chemicals

L-phenylephrine hydrochloride (PE), acetylcholine chloride (ACh), angiotensin I, anigiotensin II, Captopril, Losartan and Bay K8644 were bought from Sigma-Aldrich (St. Louis, MO, USA). The Guinep extract was dissolved in a physiological solution.

### 4.2. Plant Material and Extraction

The fruit pulp of the *M. bijugatus* tree was collected in September. Mr. Patrick Lewis, Department of Botany, University of the West Indies, Mona Campus (Kingston, Jamaica), made a botanical identification of the plant (voucher specimen AN 08, 10/11). The pulp was then blended and the juice was squeezed out through a strainer before being filtered to remove any remaining pulp fibers. Finally, the juice was placed inside a Freeze Dry machine (Freezone 4.5 L, Labconco, Kansas, MO, USA).

### 4.3. Animals

The study was conducted in accordance with the Animal Scientific Procedures Act of 1986, following approval from the University of the West Indies (UWI) Faculty of Medical Sciences (FMS) Ethics Committee (AN 06, 15/16). Seven male Sprague-Dawley rats, aged 8 to 10 weeks and weighing between 170 g and 200 g, were selected for the study. The rats were housed in plastic cages at the UWI Mona campus Animal House, where the temperature was maintained between 22 °C and 25 °C, with humidity levels ranging from 45% to 51%. Fresh tap water was provided through a bottle, and food was available ad libitum.

### 4.4. Vascular Reactivity Experiments

The animals were euthanized using cervical dislocation. Aortic rings were prepared as previously described [35] and subsequently placed in a wire myograph containing Krebs-Ringer bicarbonate (KRB) solution (in mM): 4.2 KCl, 1.19 KH_2_PO_4_, 120 NaCl, 25 NaHCO_3_, 1.2 MgSO_4_, 1.3 CaCl_2_, and 5 D-glucose, pH 7.4, 37 °C, 95% O_2_ and 5% CO_2_. Following a 30 min equilibration period, the aortic rings were stabilized by inducing three successive submaximal contractions using 60 mM KCl, with each contraction lasting 10 min. The integrity of the vascular endothelium was evaluated by applying 10 μM acetylcholine (ACh). A passive tension of 1.0 g was maintained on the aorta. Vascular contractile response was assessed by applying phenylephrine (PE) in the presence or absence of vasoactive substances, including the nitric oxide synthase inhibitor (L-NAME; 100 mM) and soluble guanylate cyclase inhibitor (1H-[1,2,4]oxadiazolo [4,3-a]quinoxalin-1-one; ODQ; 1 μM), potassium channel blockers: tetraethylammonium (TEA; 1 mM), BaCl_2_ (10 μM), 4-aminopiridine (4-AP; 1 mM), and glibenclamide (10 μM).

### 4.5. Protein and Ligand Preparation

Molecular docking analyses were conducted following validated protocols described by Saddala et al. (2020), and Logan et al. (2024), with standard modifications [24,36]. The crystal structures of human Angiotensin-Converting Enzyme (ACE, PDB ID: 1O86) [37], Angiotensin II Type 1 Receptor (AT1R, PDB ID: 4ZUD) [38], and Voltage-Gated Calcium Channel (VGCC, PDB ID: 8WE8) [39] were retrieved from the RCSB Protein Data Bank https://www.rcsb.org (accessed on 15 September 2025). Structural preparation involved the removal of all crystallographic water molecules using AutoDock Tools (MGLTools, version v1.5.7), consistent with many nutraceutical docking workflows where water-bridging effects are instead inferred from the literature. Polar hydrogens were added where applicable, and essential cofactors Zn^2+^ (ACE) and Ca^2+^ (VGCC) were retained in the active sites to maintain physiological relevance.

### 4.6. Molecular Docking

Reference and Test Ligands: Reference antagonists were selected for comparative docking validation: ACE: Captopril (PubChem CID: 44093) and Lisinopril (CID: 5362119), AT1R: Losartan (CID: 3961), VGCC: Amlodipine (CID: 2162), Nifedipine (CID: 4485), Verapamil (CID: 2520), and Diltiazem (CID: 39186). Phytochemical ligands, including Isorhamnetin-3-O-glucoside and 3-O-Caffeoylquinic acid, were identified via High-Resolution UHPLC-Q Orbitrap Mass Spectrometry and retrieved from PubChem for docking. All ligands were geometry-optimized using the MMFF94 force field in Avogadro (version 1.2.0, Open Babel backend v2.3.90) with a maximum of 10,000 steps and early convergence enabled. The optimized structures were then converted into PDBQT format using AutoDockTools (MGLTools, version v1.5.7), where Gasteiger charges were assigned and torsional degrees of freedom defined for docking.

### 4.7. Docking Validation

The docking protocol was validated by redocking the co-crystallized ligands into their respective binding sites: lisinopril with ACE (PDB: 1O86), Olmesartan with AT1R (PDB: 4ZUD), and amlodipine with VGCC (PDB: 8WE8). Grid boxes were centered on the crystallographic ligand centroid and restricted to the catalytic/orthosteric pockets. Docking was performed using AutoDock Vina 1.x (The Scripps Research Institute) (exhaustiveness = 64, num_modes = 20, energy_range = 4, seed = 1234) [40]. RMSD values (heavy atoms) between redocked and crystallographic poses were computed in PyMOL (v. 3.1), with RMSD < 2.0 Å taken as acceptable validation.

### 4.8. Docking Protocol

Docking simulations were performed using AutoDock Vina 1.x (The Scripps Research Institute, La Jolla, CA, USA) (exhaustiveness = 64, num_modes = 20, energy_range = 4, seed = 1234) [40] employing a free-energy scoring function to evaluate ligand-binding conformations. For each target, docking grids were centered on the centroid of the crystallographic ligand to restrict sampling to the validated binding pocket. Grid dimensions were calculated based on the spatial extent of the native ligand plus a 2–3 Å margin to ensure full coverage of the pocket. For ACE (PDB: 1O86), the grid was centered on lisinopril at (x = 41.036, y = 34.303, z = 46.337) with dimensions 20 × 20 × 20 Å, covering the Zn^2+^ catalytic pocket. For AT1R (PDB: 4ZUD), the grid was centered on Olmesartan at (x = –40.902, y = 63.316, z = 28.118) with dimensions 22 × 22 × 22 Å, encompassing the orthosteric site. For VGCC (PDB: 8WE8), the grid was centered on amlodipine at (x = 150.302, y = 168.056, z = 151.407) with dimensions 22 × 22 × 22 Å, restricted to the dihydropyridine (DHP) cavity and excluding non-pharmacological regions. Each ligand was docked independently into each receptor. For every docking run, multiple poses were generated, and the best-ranked pose was selected based on binding energy (ΔG) and key molecular interactions within the active site.

### 4.9. Prediction of ADMET Pharmacokinetic Properties

The pharmacokinetic properties of the compounds identified in the Guinep extract were calculated using the pkCSM server, freely available at http://biosig.lab.uq.edu.au/pkcsm/prediction (accessed 3 July 2025) [18]. SMILES structures were entered to predict various pharmacokinetic parameters related to the absorption, distribution, metabolism, excretion, and toxicity (ADMET) of the metabolites identified in Guinep extract.

### 4.10. Prediction of Biological Activity Based on Spectra

The prediction of the biological activity of the metabolites present in extracts of Guinep was performed by spectral analysis using the PASS server, freely available at https://www.way2drug.com/PassOnline (accessed 23 July 2025). The activity spectrum of a molecule provides a list of biological activities along with the probabilities of the compound being active (Pa) or inactive (Pi), with values ranging from 0 to 1 [41].

## 5. Conclusions

Together, these findings demonstrate that *M. bijugatus* possesses significant cardiovascular protective potential mediated through multitarget interactions with ACE, AT1R, and VGCC. By integrating vascular reactivity data with molecular docking and ADMET modeling, this study provides a comprehensive mechanistic framework and supports the development of Guinep-derived phytochemicals as novel, natural antihypertensive agents.

## Figures and Tables

**Table 1:** Comparison of the top scoring affinity values and bond formations for Isorhamnetin-3-O-glucoside and 3-O-Caffeoylquinic acid obtained from High Resolution UHPLCQ—Orbitrap Mass Spectrometry Guinep (*M. bijugatus*) using the ACE (PDB: 1O86).

Ligand	Affinity (kcal/mol)	Number of H Bonds	Amino Acid Residues
* Lisinopril	−8.2	4	Tyr523, Ala354, Glu384, His513
* Captopril	−5.6	3	Gln281, Lys511, Tyr520
3-O-Caffeoylquinic acid	−8.4	7	Asp415, Tyr520, His513, His353, Ala354, Glu162, Gln281
Isorhamnetin-3-O-glucoside	−9.2	7	Asp453, Lys454, Ala354, Gln281, Lys511, Asn277, Thr282

* Lisinopril and Captopril—Used as positive controls. Inhibitors of Angiotensin Converting Enzyme 1 (ACE1) and is used in the treatment of hypertension. NOTE: The more negative the Affinity (kcal/mol), the more stable and favorable the ligand–protein complex.

**Table 2:** Comparison of the top scoring affinity values and bond formations for Isorhamnetin-3-O-glucoside and 3-O-Caffeoylquinic acid obtained from High Resolution UHPLCQ-Orbitrap Mass Spectrometry Guinep (*M. bijugatus*) using the AT1R (PDB: 4ZUD).

Ligand	Affinity (kcal/mol)	Number of H Bonds	Amino Acid Residues
* Olmesartan	−8.8	1	Thr88
* Losartan	−8.8	3	Cys180, Tyr87, Thr88
3-O-Caffeoylquinic acid	−7.5	5	Trp84, Tyr88, Asp281, Ala21, Arg167
Isorhamnetin-3-O-glucoside	−8.2	5	Tyr184, Asp263, Thr88, Tyr35, Arg 167

* Losartan—Used as a positive control. AT1R receptor antagonist. NOTE: The more negative the Affinity (kcal/mol), the more stable and favorable the ligand–protein complex.

**Table 3:** Comparison of the top scoring affinity values and bond formations for Isorhamnetin-3-O-glucoside and 3-O-Caffeoylquinic acid obtained from High Resolution UHPLCQ-Orbitrap Mass Spectrometry Guinep (*M. bijugatus*) using the VGCC (PDB: 8WE8).

Ligand	Affinity (kcal/mol)	Number of H Bonds	Amino Acid Residues
* Amlodipine	−6.4	-	-
* Nifedipine	−6.9	-	-
* Verapamil	−7.5	2	Thr1056, Ser1132
* Diltiazem	−8.1	-	-
3-O-Caffeoylquinic acid	−7.7	5	Tyr1169, Ser1132, Met1509, Thr1057, Gln1060
Isorhamnetin-3-O-glucoside	−8.7	3	Thr1057, Ser1132, Met1509

* Used as a positive control as VGCC antagonist. NOTE: The more negative the Affinity (kcal/mol), the more stable and favorable the ligand–protein complex.

**Table 4:** ADMET properties of identified compounds of Guinep (*M. bijugatus*) fruit pulp extract and ACE inhibitor drugs.

Compound	Absorption		Distribution			Metabolism	Excretion	Toxicity		
	Caco2	AGI	VDss	BBB	CNS	CYP2D6/CYP3A4 Inhibitor	CT	Tox. Oral Acute in Rats (LD_50_)	Tox. Oral Chronicle in Rats (LOAEL)	Hepatox.
Isorhamnetin-3-O-glucoside	−0.55	49.86	−1.51	−1.69	−4.09	No/Yes	0.59	1.77	2.14	No
3-O-Caffeoylquinic acid	−0.51	31.03	−1.32	−1.38	−4.01	No/Yes	0.32	1.51	1.80	No
Salicylic acid glucoside	−0.43	29.19	−1.21	−1.49	−4.72	No/No	0.32	2.31	3.67	No
Aflavarin	0.44	92.22	−1.27	−1.06	−2.93	No/Yes	0.92	2.45	1.91	No
Coumaric acid glucoside	−0.37	35.02	−1.17	−1.16	−3.96	No/No	0.30	1.46	1.85	No
Feruloyl glucoside	−0.40	50.32	−1.00	−1.30	−4.07	No/Yes	0.18	1.49	1.87	No
Captopril	1.16	81.05	−0.97	−0.26	−3.09	No/No	0.30	2.38	1.21	No
Lisinopril	−0.19	21.22	0.17	−1.10	−3.73	No/Yes	0.33	1.78	1.90	Yes

Caco2: Absorption and permeability in Caco-2 cells of colon adenocarcinoma (log Papp at 10^−^^6^) cm/s). AGI: Gastrointestinal absorption (%Absorption). VDss: Steady-state distribution volume (log L/Kg). BBB: Blood–brain barrier patency (log BB). CNS: Central nervous system (log PS). CT: Total clearance (log ml/min/Kg). LD_50_: Lethal dose 50% (mol/Kg). LOAEL: Lowest level of adverse effects observed (log mg/kg/weight/day), Hepatox.: Hepatotoxicity.

**Table 5:** Results of PASS prediction for the compounds identified from the Guinep fruit pulp extract and reference drugs.

Compounds	Biological Activity															
	Antihypertensive		Cardioprotective		Vasoprotectective		Coronary Vasodilator		Peripheral Vasodilator		Calcium Regulator		Calcium Channel Blocker		Angiotensin Antagonist	
	Pa	Pi	Pa	Pi	Pa	Pi	Pa	Pi	Pa	Pi	Pa	Pi	Pa	Pi	Pa	Pi
Isorhamnetin-3-O-glucoside	-	-	0.988	0.001	0.942	0.002	0.666	0.009	0.435	0.063	0.354	0.064	-	-	-	-
3-O-Caffeoylquinic acid	-	-	0.238	0.139	0.442	0.059	0.281	0.139	0.32	0.129	0.58	0.006	-	-	-	-
Salicylic acid glucoside	-	-	0.698	0.004	0.849	0.004	0.464	0.033	0.57	0.028	0.375	0.053	-	-	-	-
Aflavarin	-	-	0.32	0.069	-	-	0.357	0.079	-	-	0.317	0.093	-	-	-	-
Coumaric acid glucoside	-	-	0.633	0.005	0.934	0.002	0.497	0.026	0.696	0.01	0.405	0.04	-	-	-	-
Feruloyl glucoside	-	-	0.652	0.005	0.905	0.003	0.661	0.01	0.724	0.008	0.385	0.048	-	-	-	-
AML	0.565	0.013	0.308	0.077	-	-	0.421	0.046	-	-	0.423	0.034	0.663	0.002	-	-
NIF	0.61	0.01	0.358	0.044	-	-	0.569	0.017	0.654	0.014	0.469	0.02	0.548	0.003	-	-
CAP	0.559	0.014	-	-	-	-	0.661	0.01			0.246	0.211	-	-	0.592	0.002
DIL	0.928	0.004	-	-	-	-	0.871	0.004			0.424	0.033	0.817	0.001	-	-
LSA	0.61	0.01	0.334	0.059	-	-	-	-	-	-	-	-	-	-	0.995	0.001
VE	0.785	0.005	-	-	-	-	0.858	0.004	-	-	0.539	0.008	0.608	0.002	-	-

Pa: Probability of activity, Pi: Probability of inactivity, AML: amlodipine, NIF: nifedipine, CAP: captopril, DIL: diltiazem, LSA: losartan, VE: verapamil.

## Data Availability

All data generated or analyzed during this study are included in this published article.
